# Corazonin Neurons Function in Sexually Dimorphic Circuitry That Shape Behavioral Responses to Stress in *Drosophila*

**DOI:** 10.1371/journal.pone.0009141

**Published:** 2010-02-10

**Authors:** Yan Zhao, Colin A. Bretz, Shane A. Hawksworth, Jay Hirsh, Erik C. Johnson

**Affiliations:** 1 Department of Biology, Wake Forest University, Winston-Salem, North Carolina, United States of America; 2 Department of Biology, University of Virginia, Charlottesville, Virginia, United States of America; Yale School of Medicine, United States of America

## Abstract

All organisms are confronted with dynamic environmental changes that challenge homeostasis, which is the operational definition of stress. Stress produces adaptive behavioral and physiological responses, which, in the Metazoa, are mediated through the actions of various hormones. Based on its associated phenotypes and its expression profiles, a candidate stress hormone in *Drosophila* is the corazonin neuropeptide. We evaluated the potential roles of corazonin in mediating stress-related changes in target behaviors and physiologies through genetic alteration of corazonin neuronal excitability. Ablation of corazonin neurons confers resistance to metabolic, osmotic, and oxidative stress, as measured by survival. Silencing and activation of corazonin neurons lead to differential lifespan under stress, and these effects showed a strong dependence on sex. Additionally, altered corazonin neuron physiology leads to fundamental differences in locomotor activity, and these effects were also sex-dependent. The dynamics of altered locomotor behavior accompanying stress was likewise altered in flies with altered corazonin neuronal function. We report that corazonin transcript expression is altered under starvation and osmotic stress, and that triglyceride and dopamine levels are equally impacted in corazonin neuronal alterations and these phenotypes similarly show significant sexual dimorphisms. Notably, these sexual dimorphisms map to corazonin neurons. These results underscore the importance of central peptidergic processing within the context of stress and place corazonin signaling as a critical feature of neuroendocrine events that shape stress responses and may underlie the inherent sexual dimorphic differences in stress responses.

## Introduction

Stress is defined as any challenge to the maintenance of homeostasis that produces adaptive behavioral and/or physiological responses [1]. Dynamic environments place significant constraints upon living systems to adapt and meet these challenges. There is significant central processing of these responses to stress, allowing for differential controls targeting different behavioral and physiological outputs. In mammals, one transmitter linked to central processing of stress is the corticotropin releasing factor (CRF). Levels of CRF transcript increase in response to many different types of stressors [2]. A major action of CRF is the control of ACTH secretion, which in turn increases the production of cortisol, which produces a multiplicity of different responses dependent upon cellular identity [3]. Another prominent action of CRF is the stress-induced inhibition of Gonadotropin releasing hormone (GnRH) signaling [4].

The corazonin neuropeptide was originally discovered based on its potent cardioacceleratory actions in the cockroach, *Periplaneta* [5]. Provocatively, in the locusts *Locusta migratoria* and *Shistocerca gregaria*, this hormone has been found to be the causal agent underlying gregarious phase pigmentation [6]. Additionally, the corazonin peptide has been shown to be co-expressed in clock cells in *Manduca sexta*, [7] and also in this same insect, coordinates the onset of the ecdysial behavioral program [8]. So, while it is recognized that corazonin is a highly conserved neuropeptide throughout the insects, a unifying theme to connect these different functions associated with corazonin has been lacking.

In *Drosophila*, cells that express corazonin also express receptor molecules for two unrelated diuretic hormones, DH_44_ and DH_31_ [9]. The significance of this finding is that, by virtue of receptor similarities, DH_44_ and DH_31_ are related to CRF and CGRP (Calcitonin-Gene Related Peptide) respectively, both of which are established mammalian stress hormones [10]–[11]. Furthermore, the corazonin neuropeptide binds to a receptor belonging to the GnRH receptor family [12]–[13]. In the mammalian hypothalamus, GnRH cells express the receptors for both CRF and CGRP and are involved in the stress-induced suppression of GnRH release [4].

We reasoned that these disparate phenotypes of corazonin might be unified under the umbrella of a stress response pathway. For example, changes in locust pigmentation during gregarious conditions are thought to be a consequence of nutrient deprivation [14]. The ecdysis behavioral program is a critical developmental period that is dependent upon appropriate water and nutrient balance, and likewise, elevated cardiac activity is a hallmark response to a variety of physiological stresses in a variety of organisms as is a circadian regulation of stress hormones [15]. Indeed, during the revision of this manuscript, two review papers hypothesized a similar role of corazonin functioning within the physiology of stress responses [16]–[17]. Additionally, the parallels between mammalian GnRH and corazonin, suggest that corazonin may be modulated by stress hormones and consequently, may represent an important substrate in the behavioral and physiological responses to stress. We report herein that different manipulations of corazonin neuronal function in *Drosophila* are manifest as differences in lifespan under different stresses. We find these differences are sex dependent and that a component of the dimorphic aspects is localized to corazonin neurons.

## Materials and Methods

### *Drosophila* Husbandry

All *Drosophila* stocks were reared on a standard cornmeal-malt-agar-molasses medium that was supplemented with proprionic acid. Stocks were housed in uncrowded conditions at 25 C on a 12∶12 LD cycle. The following stocks were used in the study: pdf-GAL4 kindly donated by Paul Taghert, Crz-GAL4 (on chromosome II) kindly donated by Jae Park, UAS-ork, UAS-rpr, UAS-NaChBac (on chromosome III), UAS-CD8-GFP (on chromosome II), UAS-tra^F^ (on chromosome II), and *w^1118^* from the Bloomington stock center. All transgenes were backcrossed in the *w^1118^* background.

### Lifespan Measurements

Measurements of lifespan have been widely employed in *Drosophila* as a metric of stress sensitivity [*e.g*, 18]. We placed 30 three to ten day old flies (males and females housed separately) in vials with a solution containing the source of the stress. We had previously determined that survivorship under the physiological stressors employed in this study was similar across this age transect (Figure S1). Animals were collected under mild CO_2_ anesthesia and placed in a vial containing the stress at ZT0 (lights on) following three days of entrainment to a 12∶12 LD cycle. A two percent agar solution was used to starve the animals, food containing 0.6 M NaCl was used to induce osmotic stress, and food at a final concentration of 20 mM paraquat was used for assessment of responses to oxidative stress. For each vial, we assessed median survival (the time of death for 50% of the population) employing non-linear regression analysis (GraphPad Prism) to calculate a mean median survival and then employed a one-way ANOVA with post-hoc Tukey's comparison for differences between genotypes/treatments. Mated males and females were housed independently and mortality was assessed every twelve hours.

### Locomotor Measurements

Locomotor activity was monitored with a TriKinetics Locomotor Monitor (Waltham, MA) on the aggregate population of thirty 3−10 day old flies. Flies were housed in a 12∶12 LD cycle for three days prior to the experiments. Flies were transferred to a vial containing the appropriate medium at ZT0. Total beam breaks were monitored continuously through an automated system and for duration of 48 hours, to avoid skewed measurements of dwindling populations. We subtracted out baseline locomotion from locomotion during stress for each genotype and then assessed the percent change in locomotion to isolate the amount of activity associated with the stress. We employed a one-way ANOVA followed by post-hoc Tukey comparisons for differences in this activity parameter between genotypes.

### Immunocytochemistry

Larval and adult progeny from flies carrying the *Crz-GAL4* transgene crossed to the UAS-CD8-GFP lines were dissected. Brains were fixed in a 4% Paraformaldehyde 7% picric acid fixative for one hour at room temperature and washed six times with phosphate buffered saline (PBS) containing Triton-X 100. A 1∶1000 dilution of anti-rabbit corazonin, a generous gift from Jan Veenstra, was incubated overnight at 4 C. Brains were washed and a Cy-3 conjugated anti-rabbit secondary antibody was applied overnight at 1∶1000 dilution. Tissues were then mounted and viewed on a Zeiss confocal microscope. All images were collected using the same settings and confocal protocol. Images were imported into Image ProPlus for quantification of immunosignals. This was done by counting the pixel intensity of corazonin immunosignals and expressing this as a ratio of autofluorescence of a similar size region of the brain using a different channel.

### Triglyceride Measurements

Total triglyceride levels were assessed through use of a Serum Total Triglyceride Kit (Sigma, St. Louis) according to manufacturer's instructions. Thirty flies were weighed, and homogenized completely in 1 mL 100 mM Tris-HCl (pH 7.4), 1 NaCl, 1% TritonX-100 buffer. Homogenates were centrifuged for 2 minutes at room temperature at 13000×g to collect particulates. One hundred microliters of supernatant was transferred to a 96 well plate and an equal volume of working triglyceride reagent was added to each well. Triplicate wells were performed for each sample and three replicate samples of flies were run for each treatment condition. Absorbance at 510 nm wavelength was measured on a Perkin Elmer Victor III multilabel microplate reader. Absorbance values were compared to standards and normalized by fly weight. A homogeneity of slopes test was performed on linear regression to compare triglyceride levels of different genotypes following starvation conditions.

### Dopamine Measurements

Dopamine levels were measured *via* HPLC as previously described [19] and through a receptor bioassay. For HPLC analysis, five brains from adults aged 3−10 days old were dissected in calcium-free ringers and placed directly into 100 µL of ice-cold 50 mM citrate acetate buffer, and homogenized completely. Extraction of adult hemolymph was done by piercing the cuticle on the thorax and abdomen and spinning for thirty seconds at 2000×G in a tube with holes to allow passage of liquid components and collection of particulates. This procedure was carried out on ninety individual flies per sample, and three samples for each sex and each genotype was collected. Two microliters of hemolymph was diluted in 100 uL of citrate buffer. Homogenates were then spun through a 0.22 micron spin filter (Millipore Corporation, Bedford, MA) prior to analysis. Samples were loaded onto a HPLC system. Dopamine levels were quantified and analyzed using Jasco ChromPass chromatography software and compared to standards. Dopamine appeared as a unitary and well-resolved peak when detected at an oxidation potential of +500 mv relative to an Ag^+^/AgCl reference electrode. This peak was confirmed as dopamine by co-migration with an authentic DA standard, and by electrochemical analysis of the half-oxidation potential. Potential statistical differences between genotypes were assessed by employing ANOVA (GraphPad, Prism).

### Corazonin Transcript Analysis

Three replicate vials of thirty flies were subjected to stress as just described. Flies were then quickly frozen and were homogenized in 1 mL of TriZol (Invitrogen, Ca). RNA was isolated according to manufacturer's recommendations. Total RNA was extracted and used as template for cDNA synthesis using a SuperScript III Reverse Transcription Kit (Invitrogen). Specific primers for the corazonin ORF were designed to flank intervening sequences to readily distinguish if amplicons originated from cDNA versus contaminating genomic DNA. The primers for corazonin were: Forward: 5′- 3′ ACT CTA ATG GCG AGA ATG TTT TGT and Reverse: ATT GAA CGG ATA GTG GCT AAT GTT. Specific primers were also designed to amplify the housekeeping gene, *RP49*. Intensity of EtBR fluorescence was determined using a Hitachi Gene Systems equipped with a CCD camera and PCR conditions were determined to be in the linear range of amplification. Values were normalized to RP49 across samples and expressed as % of baseline, *i.e*., normal conditions.

## Results

### Manipulations of Corazonin Neurons Alter Stress Sensitivity

**Figure 1:**
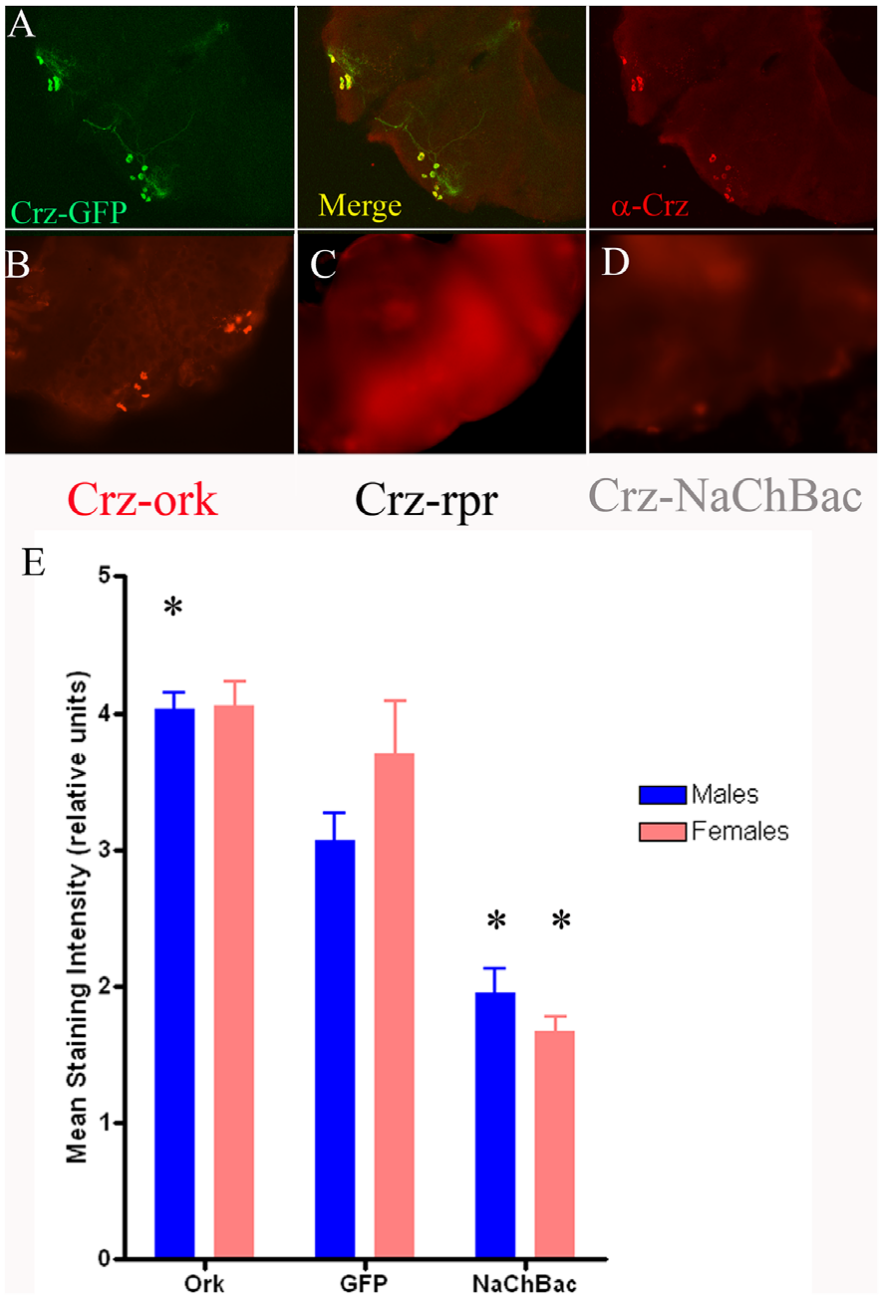
Manipulations of corazonin neurons alter levels of corazonin immunoreactivity. (**A**) Representative image of an adult brain stained with anti-corazonin expressing a GFP transgene in corazonin cells showing co-localization of immunosignals and GFP expression. (**B**) Representative image of an adult brain expressing the ork transgene. (**C**) Lack of corazonin immunosignals in brains expressing the *reaper* construct which induces apoptosis. For this image, we increased the brightness of the background to show that no cell bodies were stained with the corazonin antibody. (**D**) Representative brain from fly expressing the *NaChBac* transgene in corazonin neurons, which is aimed to facilitate peptide secretion, and note less intense staining in the soma of corazonin expressing neurons. (**E**). Quantification of immunosignals in each of these different treatments was performed on ten brains per genotype per sex. Images were collected under the same microscope settings. For quantification, a region of interest was drawn to include corazonin neuronal cell bodies and divided by the pixel intensity of a similar sized area of the brain lacking corazonin immunosignals. For animals expressing the *reaper* construct, there was no evidence of any corazonin immunolabels, preventing this analysis. Asterisks indicate significant differences (p<0.05) between the Crz-GFP ratios as determined by ANOVA.

We tested the possibility that corazonin neurons were part of the central circuitry mediating responses to various stressors by employing the GAL4-UAS binary system [20] to alter corazonin neuronal function and evaluate stress responses. We introduced the pro-apoptotic gene *reaper* to ablate corazonin cells using a specific corazonin-GAL4 line, thereby producing a corazonin deficient animal [9], [21]. We also introduced modified ion channel constructs, which have been used to silence and activate (Δork and NaChBac, respectively) discrete neuronal populations in *Drosophila* [22]–[24]. We used these constructs to alter excitability with the expectation of modulating subsequent corazonin peptide release. To evaluate the impact of transgene expression within corazonin neurons with respect to expected neuronal alterations, we employed an immunocytochemical evaluation of peptide quantity using a specific antibody against the corazonin neuropeptide (Figure 1A). We observed a complete loss of corazonin immunolabeling in flies expressing the reaper transgene (Figure 1C), a depletion of corazonin immunolabeling in NaChBac expressing (Figure 1D), and a slight increase in corazonin immunolabeling in ork expressing flies (Figure 1B) compared to flies expressing a GFP reporter (P<0.0001, Two-way ANOVA) (Figure 1E). Therefore, we conclude that these transgenes are producing phenotypes consistent with heightened secretion (NaChBac expressing), silenced secretion (ork-expressing), and ablated (reaper) corazonin neurons. However, we note that immunosignals derived from adult female brains expressing the ork channel in corazonin neurons did not significantly differ from females expressing GFP in corazonin neurons, which may reflect saturation of immunosignals in these two backgrounds.

**Figure 2:**
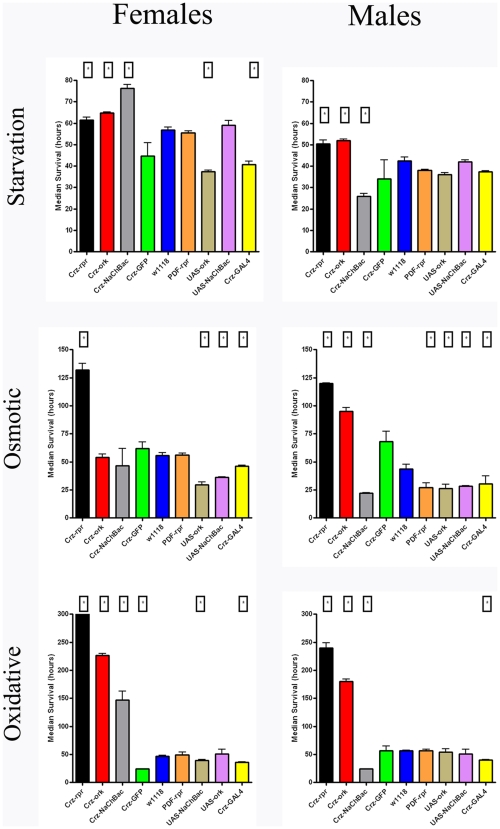
Corazonin neuronal manipulations differentially impact survival during different forms of stress. Median survival times for different genotypes subjected to three different physiological stresses. Three replicate vials of thirty individuals were assessed twice daily for survival and non-linear regression was used to estimate median survival per replicate (GraphPad Prism, San Diego). Data are mean +/− SEM from three replicates per genotype. Boxed asterisks indicate statistical difference between the background control (*w^1118^*) line (Blue bars) (ANOVA, p<0.05).

To evaluate the potential roles of corazonin neurons in mediating stress response, we first measured lifespan of these different genotypes under three different physiological stress conditions: starvation, osmotic, and oxidative. Corazonin cell ablation consistently extended lifespan during these stresses in males and females, as compared to the parental stocks or *w^1118^* genetic background controls, although the degree to which lifespan was extended varied with both gender and the nature of the stress (Figure 2). Introduction of a modified ion channel construct (Δork) to electrically silence corazonin neurons, also resulted in a lengthened lifespan under these stresses, with these effects most pronounced in males and dependent upon the nature of the stress in females. In contrast, activation of these neurons produced differential stress sensitivities in males and females. Predictably, expression of the NaChBac channel in males, results to increased stress sensitivity for all three stresses tested, as measured by lifespan. However, in females there is a strong stress by gender interaction. Notably, both the introduction of a reporter gene (UAS-CD8-GFP) to corazonin cells and the ablation of a group of well-defined neurons that express a different neuropeptide, Pigment Dispersing Factor (PDF) [25], showed stress sensitivities that were generally comparable to the *w^1118^* background controls under these heterotypic stresses (Figure 2). This indicates that changes in lifespan are likely the consequence of manipulations to corazonin neuronal populations and not solely caused by increased hybrid vigor or differences in genetic backgrounds. Although there are strong differences in quantitative traits such as lifespan [26], and some of the results we present here may be attributed to genetic background effects, our control genotypes neither showed the same large effects of the corazonin manipulations nor in many cases were background effect in the same direction. Additionally, evaluation of lifespan of genotypes with altered corazonin neuronal function indicates that the majority of these changes are a specific consequence of physiological stress, as for almost all genotypes, aging was not significantly impacted (Figure S2).

### Corazonin Neuronal Manipulations Produce Sex-Specific Effects on Locomotor Activity

**Figure 3:**
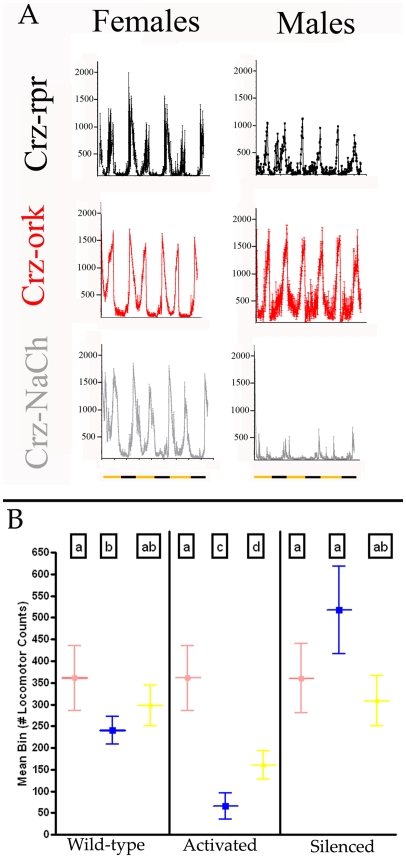
Locomotor activity is differentially affected by corazonin neuron function and sex. **A**: Locomotion was monitored for three 12∶12 LD cycles on flies expressing different transgenes in corazonin neurons. Raw data are means +/− SEM from three replicate experiments. Records begin at lights on (ZT0). **B**: Quantification of total locomotion. Mean amount of locomotion per ten minute bins were estimated for the genotypes listed plotted with 95% confidence intervals are plotted from three independent experiments. Different letters denote statistical significance from each other (Tukey post-hoc test p<0.05). Locomotor values from females are designated in red, males in blue, and for animals with feminized corazonin neurons are indicated in yellow. Animals in the first group have wild-type corazonin neuronal function (*w^1118^*), the second grouping are expressing NaChBac in corazonin neurons (Activation) and the third grouping are expressing the ork channel in corazonin neurons (Silencing).

We reasoned that the alterations in lifespan exhibited in flies differing in corazonin neuronal function may, in part, stem from changes in activity levels as activity may correlate with stress-sensitivity. We measured activity levels in flies that varied in corazonin neuronal function under a normal 12∶12 LD lighting regime. We found that the overall amount of activity levels of *w^1118^* males and females were different from each other, consistent with previous observations [27]. However, there was a significant impact on activity levels that were influenced by sex and the specific alteration to corazonin neuronal function. Specifically, males expressing the Δork channel in corazonin neurons displayed greater activity levels than *w^1118^* males. Furthermore, the opposite manipulation (NaChBac introduction) produced hypoactive males relative to *w^1118^* (Figure 3A). Both of these phenotypes were not evident in females, where both manipulations produced activity levels indistinguishable from parental genotypes (Figure S3). Ablation of corazonin neurons produced activity levels slightly lower than *w^1118^* levels for both males and females (Figure 3A). While we predicted that the ablation and silencing phenotypes would be similar for males, this inconsistent finding may be attributable to either differences in transgene impact (silencing is incomplete as compared to ablation) or developmental or physiological compensation in animals lacking corazonin neurons.

### Sexual Dimorphisms Map to Corazonin Neuronal Functions

To evaluate whether these dimorphisms were autonomous to corazonin neurons, as opposed to dimorphic downstream elements of the circuitry, we introduced the *transformer* element, which feminizes male cells [28], to corazonin neurons. Transformer expression in male corazonin cells produced intermediate amounts of locomotion between male and females, indicating a contribution of corazonin cells in the inherent dimorphisms in locomotor behaviors (Figure 3B). Based on the observations that silencing and activation of corazonin neurons produced sex-specific effects, we investigated the consequences of silencing/activation in conjunction with feminization of corazonin cells. We recombined the ork and NaChBac elements with the *transformer* element and monitored activity levels and found significant differences in the amount of locomotion that were dependent upon sex and genotype (P<0.001, F = 80.75, R^2^ = 0.9735, Two-Way ANOVA). Introduction of the transformer element along with both the ork and NaChBac elements reversed or partially reversed the effects on locomotion exhibited by males expressing either element alone (Figure 3B).

We then tested the impact of feminizing corazonin cells on survival during stress. Similarly, feminization of corazonin neurons in males bearing the NaChBac element partially reversed the stress sensitivity exhibited in males expressing the NaChBac element and tempered the phenotypes associated with male corazonin ork expression. Specifically, the median survival for silenced corazonin neurons was 51.9±0.7 hrs for starvation, 95.1±3.0 hrs for osmotic stress, and 180.0±4.1 hrs for oxidative stress. Feminization of this same genotype led to diminished effects of the silencing, with median survival being 48.0±0.2 hrs for starvation, 69.9±3.1 hrs for osmotic stress, and 132.0±0.6 for oxidative stress. Likewise, co-introduction of the *traF* with the NaChBac element lengthened survival times of the NaChBac alone (35.2±0.4 hrs as compared to 26.0±1.2 hrs for starvation, 81.7±1.1 hrs as compared to 21.9±0.6 hrs for osmotic stress, and 120.2±20.1 hrs as compared to 24.0± hrs for oxidative stress). While we acknowledge that feminization in conjunction with other corazonin neuronal manipulations do not strictly lead to the female phenotype likely reflects that feminization was limited to corazonin neurons and other dimorphic aspects of the circuitry are still male. However, the finding that any significant change is a consequence of feminization on a complex trait such as lifespan indicates an inherent dimorphism that maps to these cells, which is clear by examination of locomotor phenotypes.

### Corazonin Neurons Are Essential for Normal Stress-Induced Behavior

**Figure 4:**
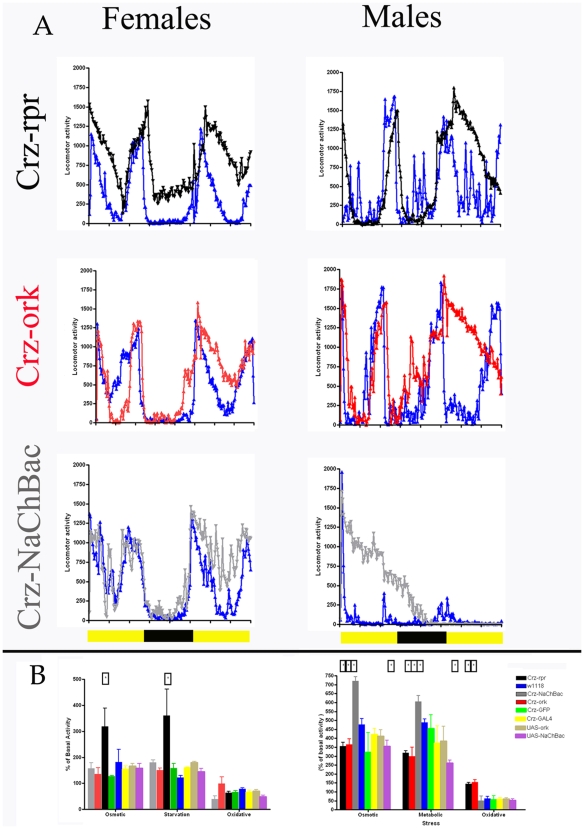
The amount of stress-induced activity differs in males and females with similar corazonin neuronal manipulations. **A.** Representative locomotor activity profiles from different populations under normal (blue) and starvation conditions (black–crz-rpr; red- crz-ork; gray–crz-NaChBac) from females (left panels) and males (right). B. Quantification of the percent of basal locomotion associated with starvation, osmotic, and oxidative stress from three replicate runs per condition (means +/− SEM). Asterisks indicate statistically significant differences from *w^1118^*.

How does the loss of a neuronal population lead to increased resistance to stress? We reasoned that one possible mechanism is that the loss of corazonin neurons might lead to critical failures in the formation of stress behaviors, thereby increasing lifespan under stress. For example, one of the many behavioral responses to starvation (and other physiological stress) is elevated activity levels [29]–[30] which ultimately causes the further loss of energy reserves and exacerbates the deleterious effects of the stress. Animals defective in producing such a behavioral response, as has recently been shown in animals deficient in the cells producing the Adipokinetic hormone, have enhanced longevity during starvation [29]–[30]. Thus, this behavioral response is counter-productive in the long-term as it leads to the further exhaustion of energetic stores, but in the short term would facilitate enhanced foraging opportunities. An alternative explanation for the changes in lifespan under stress in animals with altered corazonin neuronal function may be that secretion and/or expression of corazonin might be normally inhibited by stress, and the manipulations of corazonin neuronal excitability may either mimic or impede these normal actions of stress. To distinguish between these and other possible mechanisms, we initiated studies to evaluate specific aspects of stress response behaviors and utilized locomotor activity as a marker for such behaviors. We observed a period of hyperactivity in locomotion in *w^1118^* flies with the presentation of starvation (Figure 4A and 4B) and osmotic stress (Figure 4B), but decreased activity in flies challenged with oxidative stress (Figure 4B). In order to evaluate any potential effects of altered corazonin neuronal functions on stress-induced locomotion, we calculated the percent change of locomotion by normalizing to unstressed locomotor activity levels. Again, a strong dimorphism in this parameter was observed. Males with either silenced or ablated corazonin neurons show a diminution of stress-induced activity for starvation and osmotic stress, and are resistant to the decreases in locomotor activity caused by oxidative stress (Figure 4B). In contrast, activation of male corazonin neurons leads to enhanced activity under osmotic and starvation challenges (P<0.05, ANOVA). In females, there was also a lack of consistent effects from manipulations of corazonin neuronal excitability, although interestingly, ablation of female corazonin neurons leads to enhanced stress responses (Figure 4B), despite the observations that this alteration increases stress resistance as determined by lifespan measurements (P<0.05, ANOVA).

### Corazonin Neuronal Manipulations Alter Energy Stores

**Figure 5:**
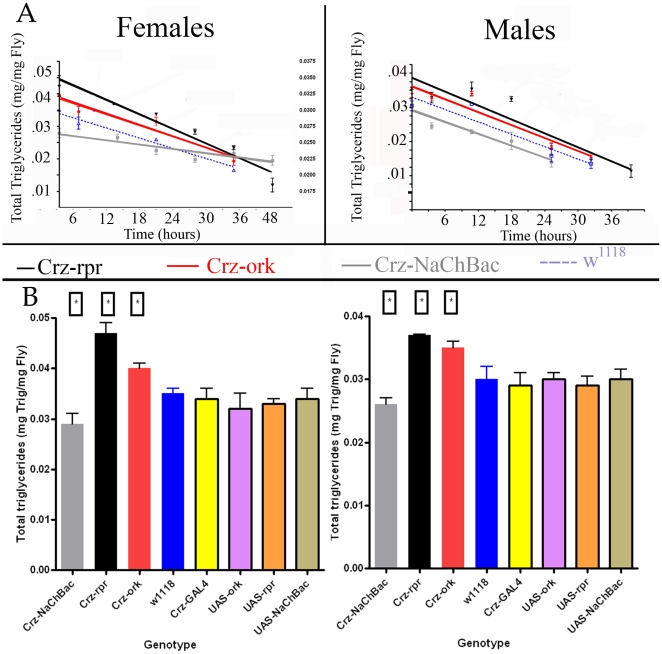
Triglyceride levels are altered in genotypes differing in corazonin neuronal function. **A.** Total triglycerides were measured in animals with different corazonin neuronal function during starvation conditions. Three replicate vials of thirty animals per genotype were placed in vials containing 2% agar, collected at the designated times and triglycerides were measured. Values were normalized to weight and compared to standards. Note that prior to starvation, corazonin neuronal ablation results in higher triglyceride content. Additionally, the slopes of the regression lines are not different in males, whereas in females, the slopes significantly differ as determined by a Homogeneity of Slopes test. **B.** Triglyceride measurements from parental and experimental genotypes under normal rearing conditions. Data are means +/− SEM from three replicate experiments and asterisks indicate statistical significance (p<0.05) (ANOVA).

Given the behavioral phenotypes associated with manipulated corazonin neuronal function, the next pertinent question is: are energy stores and/or utilization altered in these backgrounds or under stress conditions? To assess whether corazonin differentially impacted either the capacity of energy stores or its utilization, we measured total triglyceride levels in animals with different corazonin genotypes. Under normal (unstressed) conditions, levels of triglycerides vary between genotypes, but within a given genotype, there appear to be no differences between males and females. Specifically, for both sexes, ablation of corazonin neurons produced flies with the largest amount of triglycerides, followed by corazonin silenced animals, then *w^1118^* flies, and lastly flies expressing the NaChBac channel in corazonin neurons (P<0.02, ANOVA). To evaluate whether corazonin neurons alter the profile of how energy is utilized during stress, we subjected animals to starvation conditions and measured triglyceride levels. We chose to limit this analysis to starvation conditions to circumvent potential confounds of altered dietary intakes with other stresses, as dietary introduction was the method of stress-induction. In these experiments, the total amount of triglycerides decreased as a function of starvation duration for all genotypes. For comparisons of genotypes, a homogeneity of slopes test was performed following a linear regression transform. For males, this analysis revealed no differences in the slopes of the lines (F = 0.20, P = 0.81) (Figure 5A), but did reveal differences in intercept (F = 17.5, P<0.0001). This suggests that there are no differences in how triglyceride levels are mobilized or utilized during starvation, but rather the effects of genotype are limited on total triglyceride levels prior to starvation. However, for females expressing different transgenes in corazonin neurons, the slopes were significantly impacted (F = 13.8, P<0.0001). Comparing these differences in triglycerides to the changes in stress-induced lifespan suggests that these changes in energy balance may underlie the differences exhibited in stress survivorship (Figure 5B). Specifically, for males, the order of triglyceride levels matches the order of lifespan: ablated, silenced, *w^1118^*, and activated corazonin neurons. The profile of energy utilization exhibited in females with altered corazonin neuronal function also predicts stress sensitivities. Specifically, while female flies with activated corazonin neurons (NaChBac-expressing) have lower triglyceride levels, the slower turnover or utilization of these stores correlates with the longer lifespan under starvation stress.

### Levels of Dopamine Differ in Corazonin Deficient Flies

**Figure 6:**
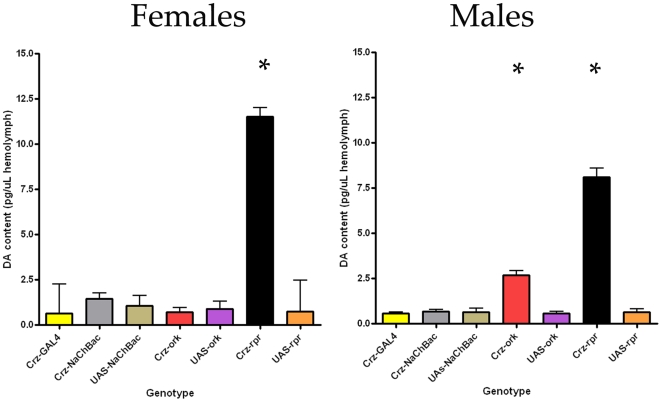
Corazonin neuronal alterations impact the levels of dopamine. Dopamine concentrations as determined by HPLC measurements on hemolymph derived from males and females with varying corazonin phenotypes. For females, only the Crz-rpr lines significantly differed in dopamine levels compared to all other control genotypes (P<0.05, Tukey's Post Test for means). In males, both the Crz-rpr and Crz-ork lines were significantly different than control parental genotypes.

Given the observation that flies with altered corazonin neuronal function differ in their hyperactivity and normal locomotor activity profiles, we investigated the possibility that corazonin may be acting in concert with other hormones to shape these behavioral responses. One candidate transmitter that may be interacting with corazonin neurons is dopamine, based on previous descriptions of altered dopamine synthesis during stress and phenotypes associated with genetic differences in dopaminergic signaling [31]–[35]. We tested the possibility that dopamine signaling may be altered in the flies with genetically altered corazonin neuronal functions. We first tested this hypothesis through employing a receptor bioassay. Larval hemolymph derived from flies with specific corazonin neuronal alterations was applied to HEK-293 cells expressing the dopamine receptor encoded by *CG9761* [36] and a luciferase reporter for cAMP [37] the second messenger through which this receptor signals. The amount of luminescence detected was greatest from hemolymph derived from flies lacking (rpr) followed by silenced (ork), *w^1118^* and activated (NaChBac) corazonin neurons (Figure S4). While such results are indicative of an interaction between corazonin function and dopamine content, this measure only provides a relative measure of dopamine, as reporter-only transfected cells exhibited greater luminescence than vehicle treated cells, and for technical reasons only allowed investigation of larval stages. Therefore, we employed HPLC measurements on adult flies with altered corazonin neuronal function to quantify the impact of corazonin on dopamine levels (Figure 6). Dopamine levels were significantly higher in hemolymph derived from both males and females with ablated corazonin neurons. However, for the other genotypes there was also a strong dependence upon sex (P<0.0001, F = 33.44 Two-Way ANOVA). For males with silenced corazonin neurons, dopamine levels were also higher than parental genotypes. However, in females both activated and silenced corazonin cells have comparable dopamine levels as compared to parental genotypes. Since manipulations predicted to alter excitability of these neurons don't result in large or consistent changes in dopamine levels (see legend to Fig. 6), these manipulations may not fully block corazonin release. Notably, these effects were seen only in hemolymph samples, as brains derived from these different genotypes had dopamine levels that were not different than *w^1118^* (data not shown). While dopamine levels change as a function of development, the most significant changes in dopamine levels occur coincident with eclosion and cuticular tanning, which are events that preceded these observations [38]. Additionally, we report similar patterns of altered dopamine levels in two different life stages, those of wandering third instar larva and adults, suggesting that altered dopamine levels are a specific consequence of corazonin neuronal manipulations.

### Corazonin Expression Decreases during Stress

**Figure 7:**
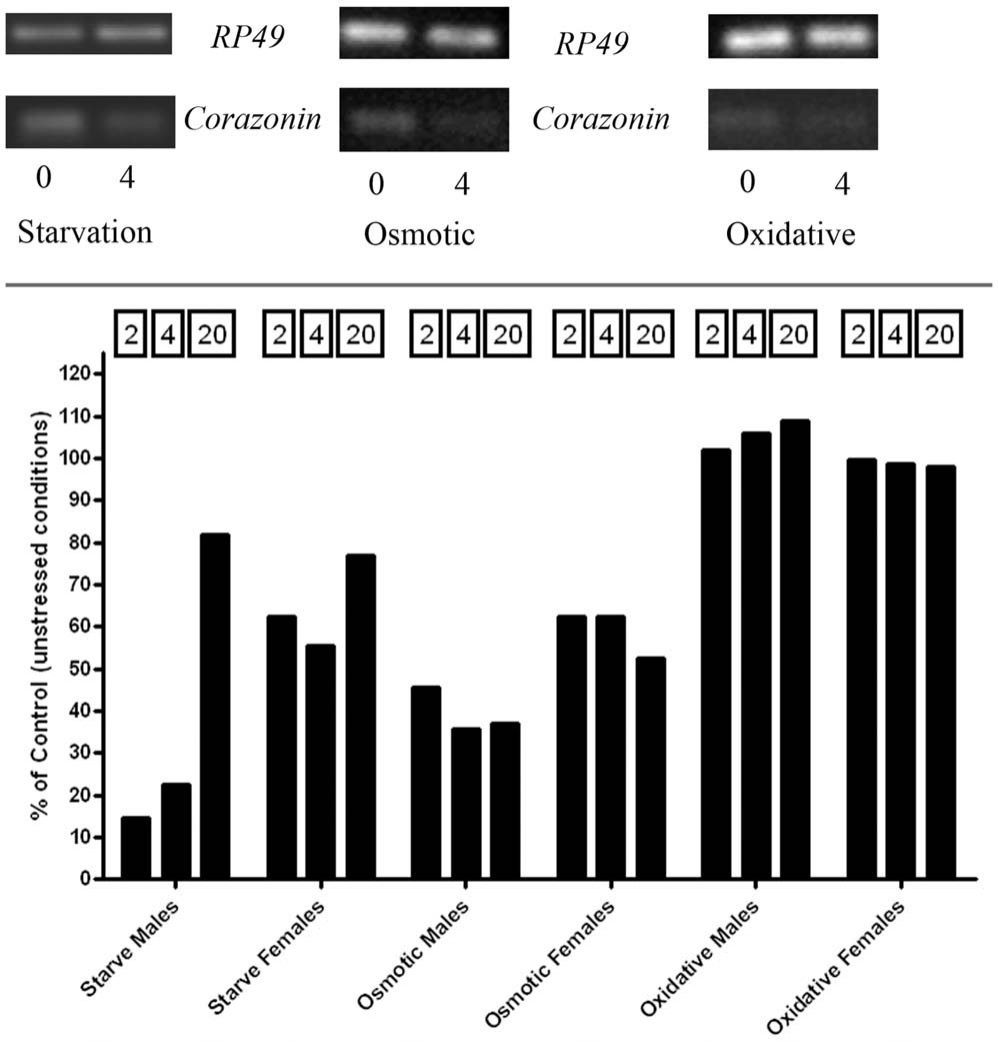
Corazonin transcript levels decrease during stress. **Top**: Representative gels from experiments of corazonin and RP49 expression in the *w^1118^* genotype under starvation, osmotic, and oxidative stress. **Bottom**: Changes in corazonin expression under different stresses for noted times. Data are represented as a % of basal (unstressed) and are normalized to RP49. Starvation and osmotic stress cause a decline in corazonin expression and rises as a function of stress duration. Oxidative stress produces no change in corazonin expression.

The above results demonstrate a requirement of corazonin neurons within multiple behavioral and physiological responses accompanying different stresses. However, a possible explanation for these phenotypic differences is that corazonin neurons participate in the developmental organization of responsible central and/or peripheral targets as opposed to acute actions of these neurons during stress. To begin to differentiate between these possibilities, we measured corazonin transcript expression during periods of stress. To gauge relative expression of the corazonin transcript, we normalized levels to those ascertained for *RP49*. These experiments show a decrease in corazonin transcript expression following starvation and osmotic stress, and such decreases were more pronounced in males (Figure 7). This rapid decline of expression levels gradually returns to normal levels as a function of starvation stress duration in males. Corazonin transcript levels were largely unaffected by oxidative stress.

## Discussion

**Figure 8:**
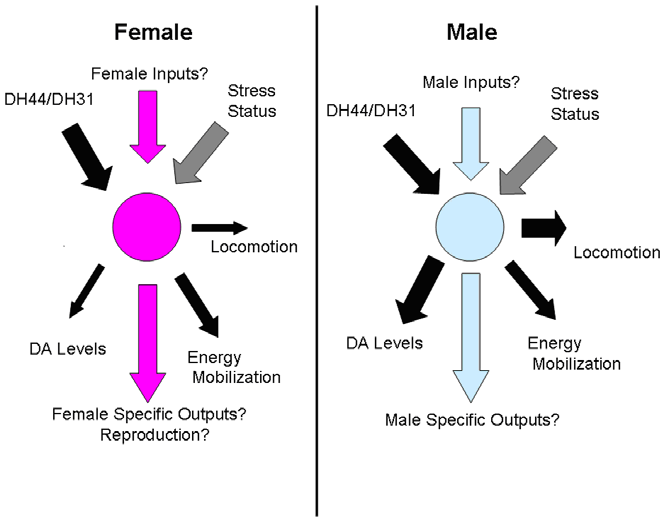
Model of corazonin neuronal function in *Drosophila*. Corazonin neurons in both males and females express the receptors for DH_44_ and DH_31_ (Johnson et al., 2005) but may also have sex-specific inputs that are currently unknown. It may be that stress status is related either through these inputs or yet unidentified inputs. Corazonin neurons participate either directly or indirectly to regulate locomotor activity, dopamine levels, and triglycerides but exhibit sex-specific differences in the extent and nature of these phenotypes. Arrow size reflects the magnitude of interactions. Additionally, it may be that there are sex-specific targets of corazonin neurons.

Our observations implicate corazonin neurons as a critical anatomical locus that participates in stress response, however, we cannot rule out the possibility that the phenotypes associated with corazonin neuronal manipulations reflect changes in an unknown co-transmitter. However, there is no other known transmitter that is co-expressed with corazonin, and previous functional descriptions of the corazonin peptide are consistent with a role in mediating stress responses. Specifically, previous analysis of the actions associated with corazonin, include acceleration of heart rate [5], association with the ecdysial behavioral program and the circadian system [7], [8], and corazonin levels are correlated with gregarious phase pigmentation in *Locusta* [6]. We suggest that these disparate phenotypes might reflect a general involvement of corazonin neurons functioning within stress responses for the reasons given previously (Figure 8). We also note that the demonstration of corazonin neuron involvement within stress response behaviors is paralleled by previous observations describing suppression of GnRH (suspected corazonin homolog, by virtue of receptor similarities) release as part of the stress response in mammals, by virtue of its modulation of stress hormones [4]. The observations that manipulations of crz neuronal physiology manifest as alterations in lifespan and other behavioral and physiological parameters during heterotypic stresses suggest a general contribution of crz neurons to stress responses.

In general, stress responses are thought to reflect a shift away from physiologies less critical for the maintenance of homeostasis during adverse environmental changes [39]. Throughout the mammals, responses to stress include inhibition of urinary, digestive, immune, and reproductive physiologies and simultaneous activation of cardiac, muscle, respiratory, and energy mobilization processes. The enhanced levels of activity are of significant selective advantage, as this would facilitate the removal of the organism from the source of stress. As mentioned previously, there is an inverse relationship between the strength of stress responses and lifespan during homeostatic challenges. For example, mobilization of energy stores to facilitate higher activity levels might lead to enhanced foraging, but also more quickly exhaust energy levels which would lead to a more rapid death in the event food was not found. In general, heightened locomotor activity would facilitate escape behaviors [1]. Corazonin signaling and its impact on activity levels suggests a complex interplay between central signaling and peripheral tissues which determine stress sensitivity. Under non-stressed conditions, our results show a strong influence on the level of corazonin signaling on activity levels in males. In contrast, there is apparently a lack of corazonin dependence in determining normal activity levels in females. However, corazonin signaling does influence the dynamics of stress-induced activity levels in both males and females. In males, the amount of locomotion is inversely related to lifespan duration, and this demonstration is expected as that the increased activity levels lead to rapid decreases in energy reserves, and also lead to abbreviated lifespan [1]. However, in females, enhanced locomotor activity is not correlated with lifespan under stress, as the ablation of female corazonin neurons leads to enhanced activity but also lengthened lifespan. Perhaps, part of the difficulties in interpreting these results is that some of the effects of altered corazonin signaling may be manifest as developmental changes of the circuitry that regulates stress behaviors. We are currently attempting to address the contributions of developmental versus acute effects of altered corazonin signaling in *Drosophila* by employing different strategies to alter corazonin signaling at restricted developmental intervals.

The demonstration that corazonin neurons alter both the size of energy stores and at least in females, also alter the utilization of energy suggests that corazonin neurons function in the mechanism of resource allocation. This observation is supported by a recent report that shows differences in the levels of trehalose, a major carbohydrate energy source in insects, in flies lacking corazonin [40]. The impact of altered corazonin neuronal functions on activity levels might also be indicative of a role for corazonin-mediated reallocation of energy during stress. The effects of altered corazonin function on normal locomotion may reflect inherent differences in metabolic capacities which in turn regulate basal activity levels.

Throughout the Metazoa, there are inherent differences in how the different sexes respond to stress [*e.g.*, 41]. Specifically, during stress, males continue to court, whereas in contrast, female receptivity is significantly lowered [31]. It could be that corazonin signaling is altering male-specific responses and has novel roles in females, or alternatively corazonin has similar roles in both sexes but the effects of which are compensated by other dimorphic aspects of the circuitry. Notably, our reports here on the functional differences in between the sexes of corazonin neuronal function are supported by a recent anatomical study that identified four corazonin-expressing neurons present only in the abdominal ganglia of adult males [40]. Furthermore, given that the corazonin receptor is ancestrally related to vertebrate GnRH receptors [42] and that GnRH signaling throughout the vertebrates is involved in dimorphic behaviors [*e.g.*, 43], the sexual dimorphisms we observed further support the hypothesis of a functional conservation between corazonin and GnRH signaling.

The differences between the actions of corazonin in the different sexes suggest fundamental differences in regulatory elements of the neural circuitry in males and females. In males, the loss of corazonin signaling produced phenotypes similar to those with the silencing of corazonin neurons, and as expected, the activation of corazonin neurons consistently produced opposite phenotypes. This suggests a simple model in males, in which the absolute amount of corazonin regulates stress behaviors and sensitivity. In contrast, the lack of corazonin signaling results in a stress-resistant phenotype, whereas the activation or silencing of these neurons results in varied phenotypes. We speculate that in females the absolute presence of corazonin may help determine stress sensitivity, since ablation of female corazonin neurons leads to changes in multiple stress physiologies and behaviors. We speculate that females are resistant to changes in the levels of corazonin by specific mechanisms regulating the timing of corazonin release in females. In support of this notion, it has been noted that the efficacy of the elements employed here (ork and NaChBac) to manipulate excitability are in part dependent upon fundamental aspects of the circuitry targeted. Specifically, these elements have been determined to be more effective in circuits in which temporal coding is not the critical component [22]–[24], [44]–[45]. If this is the case with corazonin neurons, this would suggest that there are fundamental differences in corazonin circuits between males and females, and may explain the differences we have observed utilizing this experimental strategy. Alternatively, it could be that there are some compensatory mechanisms of downstream components that may explain some of the different effects of altered corazonin neuronal function in females. However, we note that a significant contribution of the dimorphic aspects is localized to corazonin neurons, suggesting that a major component is likely to be some intrinsic differences in regulation of corazonin secretion.

The connections between corazonin and altered dopamine implicate a complex neuroendocrine circuitry that mediates behavioral and physiological responses to stress. Dopamine signaling in *Drosophila* contributes to many different behavioral and physiological processes that are targets of stress modulation. For instance, dopamine elevates heart rate [46], mediates female receptivity [47], affects general arousal state [48], alters egg laying behaviors [49], and contributes to circadian rhythmicity [50]. These various phenotypes are largely consistent with behavioral and physiological modifications in the general adaptation syndrome proposed by Selye. [1], [51]. Dopamine levels increase with the presentation of heat stress, as measured *via* high performance liquid chromatography (HPLC) on *Drosophila* heads [33]. In addition, the activity of the rate limiting enzyme in dopamine synthesis, tyrosine dehydroxylase (TH), is altered under several types of heterotypic stresses in *Drosophila* [31]–[34]. Previous studies have shown that dopaminergic neuron survival is diminished during oxidative stress [35]. Notably, the behavioral deficits accompanying the loss of dopaminergic neurons can be tempered through application of the dopamine precursor, DOPA [52]–[53]. Our results showing a strong resistance of animals lacking corazonin neurons to oxidative stress is consistent with the observations of increased dopamine levels in these same animals. Furthermore, dopamine actions are known to be dimorphic in *Drosophila*, and our results are consistent with previous observations documenting the contributions of dopamine to dimorphic behaviors [54]. Notably, the connection between dopamine and GnRH signaling in the vertebrates is well-documented [*e.g.*, 55] and lends additional support for functional parallels between mammalian GnRH and insect corazonin signaling. Corazonin may be altering dopamine levels either at the level of synthesis, degradation, or release, the latter may, in part explain the failure to discern differences in dopamine levels in the brain. Alternatively or additionally, altered dopamine levels may be selectively altered only in the periphery via corazonin neuronal manipulations. Future studies that identify the distribution of the corazonin receptor in specific regards to dopaminergic cells may assess the specific mechanisms of corazonin actions on dopamine.

Our observation of inhibition of corazonin transcript expression during starvation and osmotic stress suggests that stress reduces corazonin signaling, consistent with previous results employing microarray analysis, which also found a reduction of corazonin transcript under stress [56]. Furthermore, analysis of the ensemble of stress phenotypes also supports a model of stress-induced inhibition of corazonin signaling. We speculate that this inhibition may be caused by signaling through either the DH_31_ or DH_44_ peptide transmitters, as corazonin neurons express both of these receptors [9] and in mammals, the homologs of these peptide hormones are both involved in the stress-induced suppression of GnRH release [4]; this observation suggests that this particular neural circuit may be ancestral and functions within the mediation of stress responses.

We presume that the multiple phenotypes associated with corazonin neurons reflect multiple primary effects of altered corazonin neuropeptide secretion as well as secondary indirect actions. Clearly, hormonal signaling systems can impact multiple tissues depending on the scope of receptor distribution, as well as initiate a sequence of endocrine events via the regulation of other hormones. Further studies aimed at addressing phenotypes associated with the corazonin receptor may offer insight into the specific mechanisms of corazonin actions in regards to stress-altered behaviors.

## Supporting Information

Figure S1 Effect of age on physiological stress sensitivity. We assessed the impact that an animal's age had on starvation and osmotic stress sensitivity. We isolated thirty age-matched individuals and assessed mortality as described in text. Median survival +/− SEM was determined from three replicates and fifteen day old female flies have lower survival than 3 day old and 10 day old animals (P<0.05, ANOVA), whereas while there was a general trend in lower lifespan in males as a function of age there was no statistical difference.
(1.79 MB TIF)

Figure S2 Lifespan in animals with corazonin neuronal manipulations. To evaluate absolute lifespan, animals were placed in vials containing normal food media after being collected and co-housed for three days. Animals were subsequently sorted by sex and placed in vials with normal media in groups of thirty. Vials were changed once every three days and mortality was assessed once daily. Males with ablated corazonin neurons are long-lived as compared to w1118 or Crz-GFP (P<0.05 ANOVA), and females with ablated or silenced corazonin neurons are longer lived than w1118 (P<0.05, ANOVA), but are comparable to Crz-GFP.
(7.95 MB TIF)

Figure S3 Basal locomotion in parental and control genotypes. We measured locomotion with methods previously described. Total locomotor counts were monitored for a twenty four hour period from three replicate populations. There were no significant deviations in locomotor activity (P>0.05 ANOVA)
(9.77 MB TIF)

Figure S4 Quantification of dopamine levels of larval hemolymph. Larval hemolymph was extracted and 100 uL of hemolymph was collected from three hundred individuals. Homogenate was spun through a filter and then placed on HEK-293 cells that were transfected with either cDNA encoding the DopR gene (Feng et al., 1996) and a Cre-luc reporter (Hearn et al., 2002) or with the Cre-luc reporter alone. Cells were incubated for four hours, lysed and luminescence levels were quantified using a LucLite kit (Perkin Elmer, Waltham, MA) and luminescence counts were collected using a Victor Wallac III Multilabel Plate Reader. Luminescence levels were collected on triplicate wells, and ANOVA, Tukey post-hoc test were employed to assess statistical differences. Different letters denote statistical significance (P<0.05).
(5.11 MB TIF)
