# Supplementary figures and images for: Corazonin Neurons Function in Sexually Dimorphic Circuitry That Shape Behavioral Responses to Stress in Drosophila
Source: PLoS One. 2010 Feb 10;5(2):e9141. doi: 10.1371/journal.pone.0009141 (PMC2818717; doi:10.1371/journal.pone.0009141)

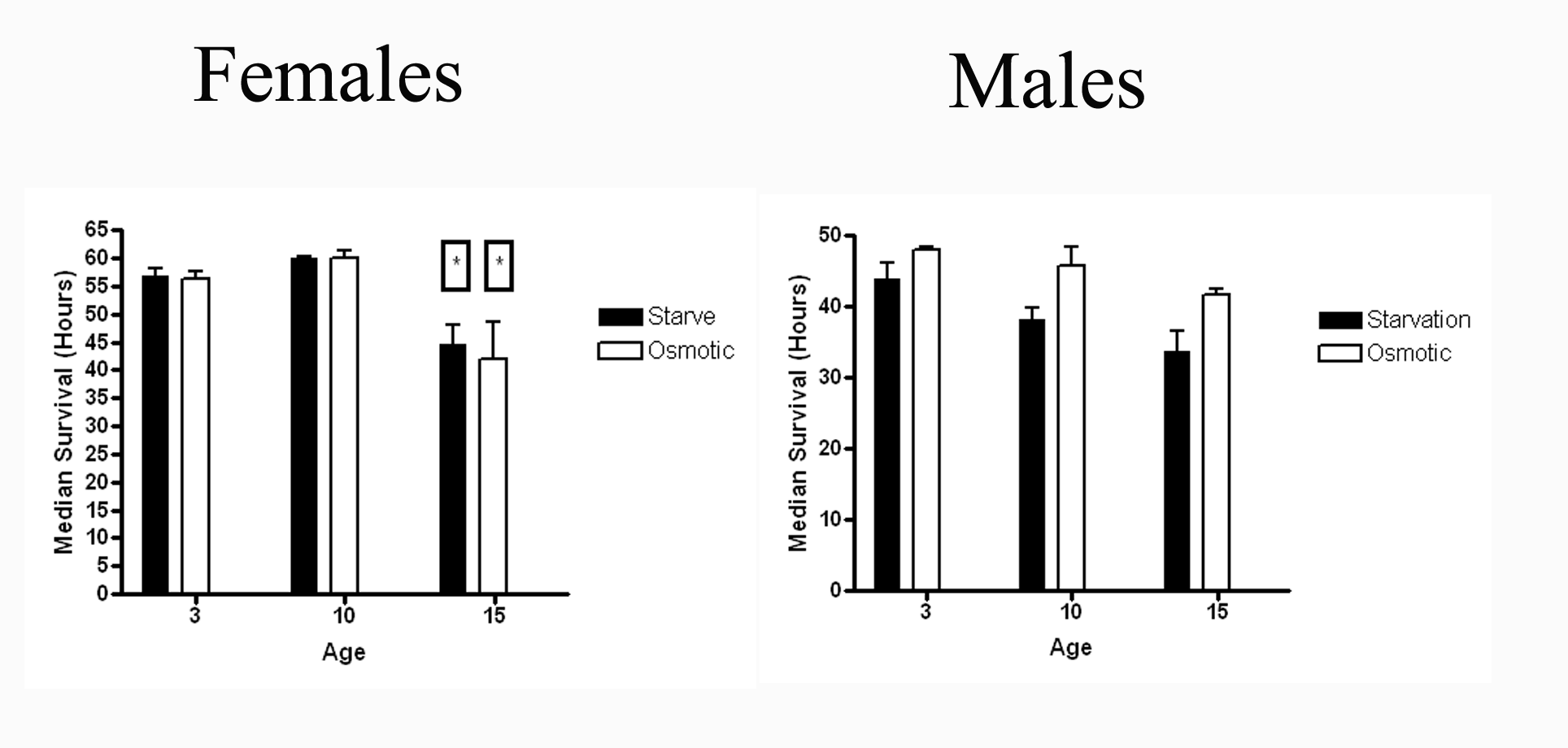

Supplement: Figure S1 — Effect of age on physiological stress sensitivity. We assessed the impact that an animal's age had on starvation and osmotic stress sensitivity. We isolated thirty age-matched individuals and assessed mortality as described in text. Median survival +/− SEM was determined from three replicates and fifteen day old female flies have lower survival than 3 day old and 10 day old animals (P<0.05, ANOVA), whereas while there was a general trend in lower lifespan in males as a function of age there was no statistical difference. (1.79 MB TIF) [file pone.0009141.s001.tif]

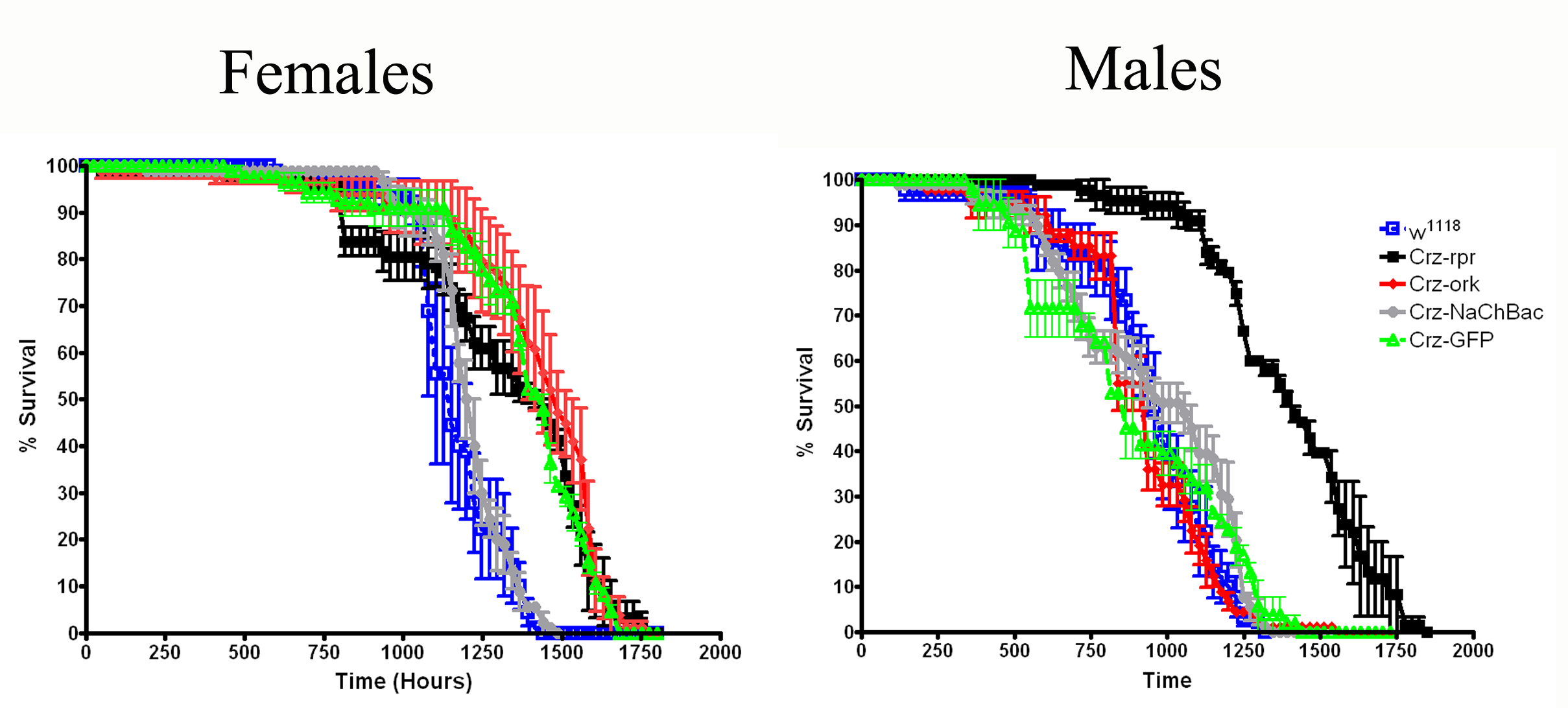

Supplement: Figure S2 — Lifespan in animals with corazonin neuronal manipulations. To evaluate absolute lifespan, animals were placed in vials containing normal food media after being collected and co-housed for three days. Animals were subsequently sorted by sex and placed in vials with normal media in groups of thirty. Vials were changed once every three days and mortality was assessed once daily. Males with ablated corazonin neurons are long-lived as compared to w1118 or Crz-GFP (P<0.05 ANOVA), and females with ablated or silenced corazonin neurons are longer lived than w1118 (P<0.05, ANOVA), but are comparable to Crz-GFP. (7.95 MB TIF) [file pone.0009141.s002.tif]

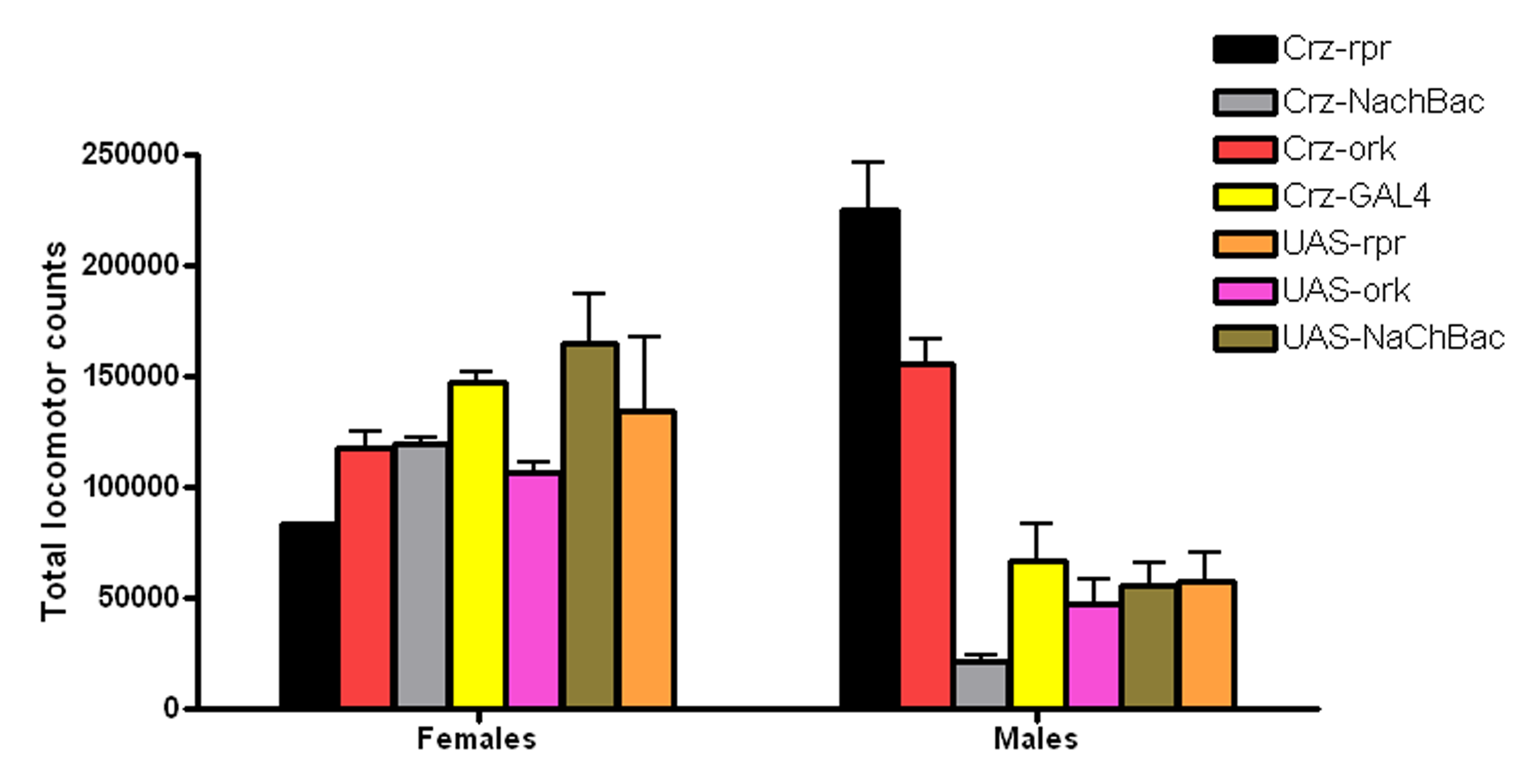

Supplement: Figure S3 — Basal locomotion in parental and control genotypes. We measured locomotion with methods previously described. Total locomotor counts were monitored for a twenty four hour period from three replicate populations. There were no significant deviations in locomotor activity (P>0.05 ANOVA) (9.77 MB TIF) [file pone.0009141.s003.tif]

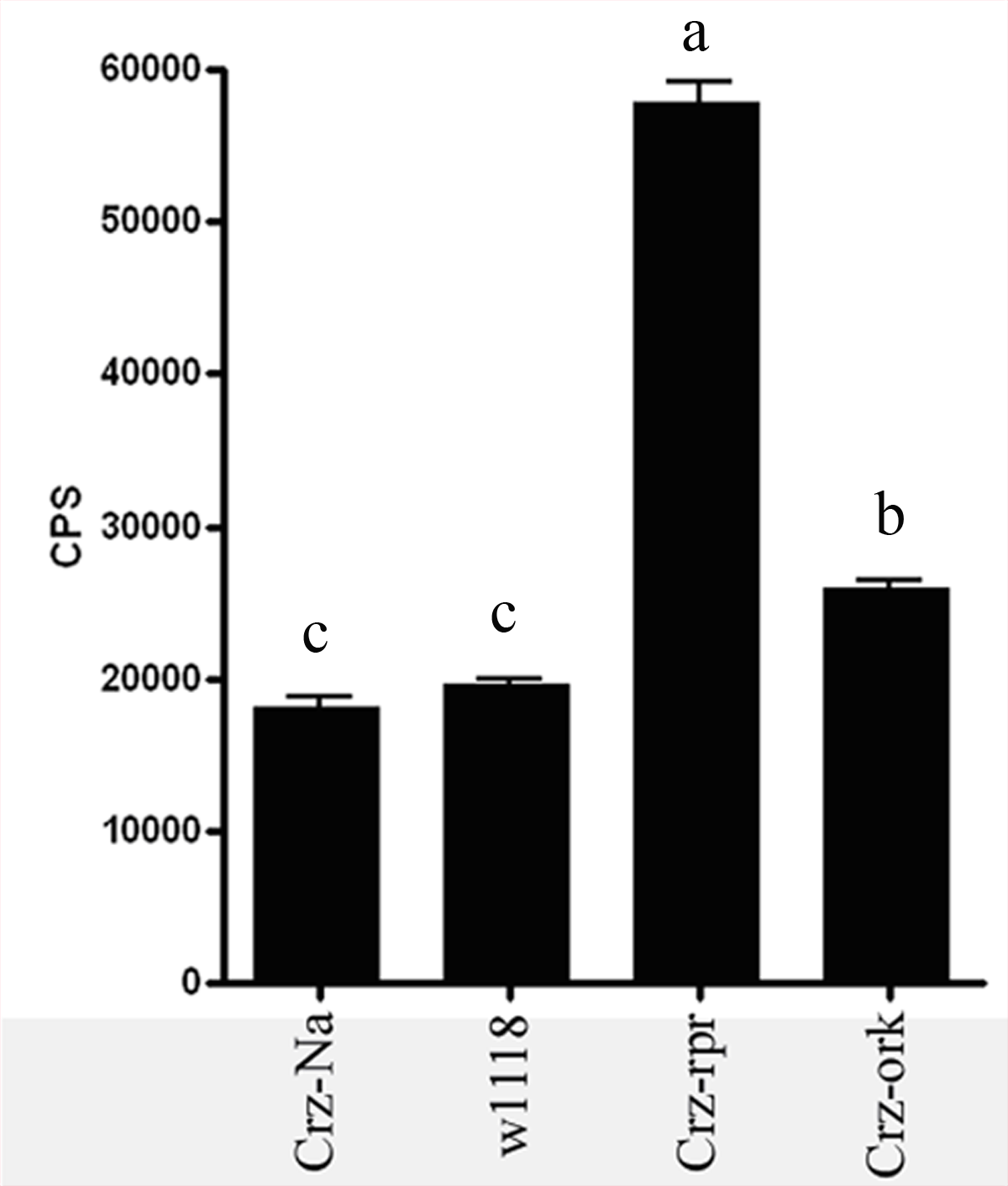

Supplement: Figure S4 — Quantification of dopamine levels of larval hemolymph. Larval hemolymph was extracted and 100 uL of hemolymph was collected from three hundred individuals. Homogenate was spun through a filter and then placed on HEK-293 cells that were transfected with either cDNA encoding the DopR gene (Feng et al., 1996) and a Cre-luc reporter (Hearn et al., 2002) or with the Cre-luc reporter alone. Cells were incubated for four hours, lysed and luminescence levels were quantified using a LucLite kit (Perkin Elmer, Waltham, MA) and luminescence counts were collected using a Victor Wallac III Multilabel Plate Reader. Luminescence levels were collected on triplicate wells, and ANOVA, Tukey post-hoc test were employed to assess statistical differences. Different letters denote statistical significance (P<0.05). (5.11 MB TIF) [file pone.0009141.s004.tif]
